# Epicardial adipose tissue as a mediator of cardiac arrhythmias

**DOI:** 10.1152/ajpheart.00565.2021

**Published:** 2021-12-10

**Authors:** Kiran Haresh Kumar Patel, Taesoon Hwang, Curtis Se Liebers, Fu Siong Ng

**Affiliations:** National Heart and Lung Institute, Imperial College London, London, United Kingdom

**Keywords:** arrhythmia, epicardial adipose tissue, inflammation, obesity

## Abstract

Obesity is associated with higher risks of cardiac arrhythmias. Although this may be partly explained by concurrent cardiometabolic ill-health, growing evidence suggests that increasing adiposity independently confers risk for arrhythmias. Among fat depots, epicardial adipose tissue (EAT) exhibits a proinflammatory secretome and, given the lack of fascial separation, has been implicated as a transducer of inflammation to the underlying myocardium. The present review explores the mechanisms underpinning adverse electrophysiological remodeling as a consequence of EAT accumulation and the consequent inflammation. We first describe the physiological and pathophysiological function of EAT and its unique secretome and subsequently discuss the evidence for ionic channel and connexin expression modulation as well as fibrotic remodeling induced by cytokines and free fatty acids that are secreted by EAT. Finally, we highlight how weight reduction and regression of EAT volume may cause reverse remodeling to ameliorate arrhythmic risk.

## INTRODUCTION

Atrial fibrillation is the most common arrhythmia globally and its impact on healthcare systems is forecast to increase with an aging population (1, 2). Although ventricular arrhythmias are comparably less common, they are nonetheless associated with significant morbidity and higher risk of mortality (3). With better understanding of the mechanisms underpinning arrhythmogenesis and ability to manage arrhythmias, there has been increasing interest in preventative measures to reduce their prevalence.

Among the cardiovascular risk factors, there has been a particular focus on adiposity and obesity in generating a proarrhythmic substrate (4). Obesity is an economically burdensome pandemic, which has been associated with increased arrhythmic risk (5). It is a multifaceted syndrome that often coexists with cardiometabolic perturbation, such as hypertension and obstructive sleep apnea, which may themselves confer increased risk of arrhythmias (6–8). Nevertheless, there is now compelling evidence linking adipose tissue volume independently with arrhythmic risk and weight loss with arrhythmia-free survival (9–11). Although obesity is characterized by excess whole body adiposity, visceral adipose tissue (VAT) has been shown to be more detrimental to health than its subcutaneous counterpart (12). Consistent with this, epicardial adipose tissue (EAT) has been implicated in arrhythmogenesis owing to its intimate relationship with the underlying myocardium (13, 14).

In this review, we first outline the classification of adipose tissue and the anatomy, physiology, and pathophysiology of epicardial adipose tissue (EAT). We then summarize the evidence for its role as a transducer of inflammation to the underlying myocardium and outline the mechanisms by which it may induce an arrhythmogenic substrate. Finally, we review the evidence for the reversibility of the proarrhythmic substrate associated with obesity with weight reduction strategies.

## EPICARDIAL ADIPOSE TISSUE: HISTOLOGY, ANATOMY, AND PHYSIOLOGY

**Figure 1.**
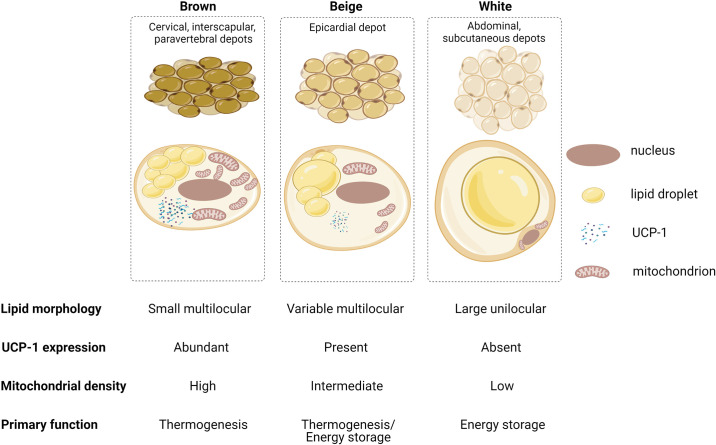
Characteristics of brown, beige, and white adipose tissue. Brown adipose tissue (BAT) is mainly located in cervical, interscapular, supraclavicular, and paravertebral regions. Brown adipocytes contain several small vacuolated lipid droplets, contain multiple mitochondria that give them characteristic color, and express uncoupling protein-1 (UCP-1) in relative abundance. These features reflect their primary function in nonshivering thermogenesis. By contrast, white adipose tissue (WAT) is more widely distributed subcutaneously and viscerally. WAT adipocytes typically contain a single large lipid droplet with few mitochondria and minimal UCP-1 and serve as an energy store. Beige adipose tissue, of which epicardial fat is example, is an intermediate phenotype characterized by multiple loculated lipid droplets with a mitochondrial density and UCP-1 expression that is greater than WAT but less than BAT. Beige adipose tissue contributes to thermogenesis as well as serving as an energy store. Created with BioRender.com, and published with permission.

Adipose tissue can be broadly categorized as white, beige, or brown (Fig. 1). White adipose tissue (WAT) is widely distributed throughout the body as either subcutaneous or visceral fat. Its main function is that of energy storage, and hence, its adipocytes consist of a single lipid droplet of triglycerides, giving it a characteristic yellow color, with minimal space for mitochondria, which may be thin, elongated, and often variable in number (15). WAT also secretes a range of hormones, cytokines, complement and growth factors that have both endocrine and paracrine effects on adjacent and distant tissues (16). On the other hand, brown adipose tissue (BAT) is commonly located in cervical, supraclavicular, axillary, paravertebral, mediastinal, and upper abdominal regions and has an antagonistic role to WAT; it dissipates energy through thermogenesis (15, 17). The cellular composition of BAT reflects its function, in that there is abundance of mitochondria within the adipocytes giving it a brown hue, and triglycerides are stored as small vacuoles. BAT expresses uncoupling protein-1 (UCP-1), which has been implicated in nonshivering thermogenesis by uncoupling mitochondrial oxidative phosphorylation. It also has a denser microcirculation and sympathetic innervation owing to a greater oxygen demand and its need to respond to thermogenesis (15). Because of this unique property, BAT is widely found in small mammals and newborns in which the high surface area-to-volume ratio predisposes them to heat loss. In adulthood, BAT has a minimal role in thermoregulation as core body temperature is regulated via other mechanisms, such as muscle shivering and insulation with WAT.

Beige adipose tissue (BeAT), also called “brite” adipocytes, is an intermediate phenotype that is characterized by WAT and BAT features. In contrast to WAT and BAT, BeAT develops postnatally and is inducible. It typically develops within the subcutaneous WAT from a distinct preadipocyte population (18) or via transdifferentiation of white adipocytes (19, 20), i.e., “browning” of WAT. Like BAT, BeAT exhibits multilocular lipid droplets and UCP-1 expression and contains a dense population of mitochondria and therefore functionally regulates energy balance and thermogenesis (Fig. 1). Wu et al. (18) showed multilocular beige adipocytes can develop from a subset of unilocular preadipocyte upon noradrenergic stimulation, characterized by an enhanced expression of thermogenic genes such as UCP-1. However, despite its similarities to BAT (Fig. 1), BeAT exhibits a unique molecular signature. This includes, for example, expression of Tbx1 and molecules involved in the lipid metabolism and inflammatory response pathways (Slc27a1 and CD137, respectively) (18).

**Figure 2.**
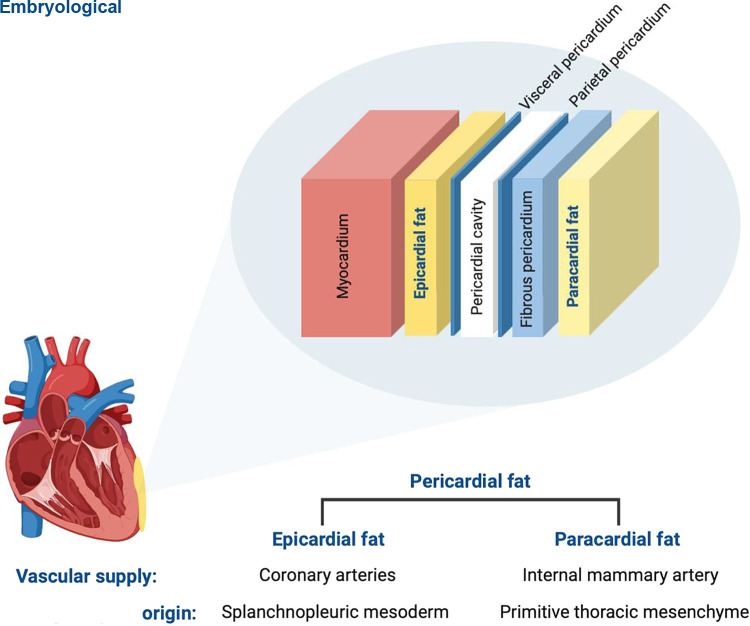
The anatomy of epicardial adipose tissue (EAT) in relation to the myocardium and surrounding structures. EAT lies deep to the visceral pericardium (i.e., the epicardium) and in direct contact with myocardial tissue. It derives from the splanchnopleuric mesoderm and draws its vascular supply from the coronary arteries. By contrast, paracardial fat refers to adipose tissue external to the parietal pericardium. Its origin is from the primitive thoracic mesenchyme and is perfused by the internal mammary artery. Pericardial fat refers to EAT and paracardial adipose tissue. Created with BioRender.com, and published with permission.

Epicardial adipose tissue (EAT) is a white visceral fat depot that uniquely exhibits BeAT features (21–23). Like BAT, EAT expresses UCP-1 and contains small multilocular adipocytes reflecting the transformation of WAT-derived precursor cells into mature thermogenic “brown-like” adipocytes (21). EAT is localized deep to the visceral pericardium, i.e., the epicardium, and therefore is in direct contact with the underlying myocardium due to a lack of a fascial boundary (Fig. 2). It can cover up to 80% of the heart surface and contributes up to 20% of the whole cardiac mass despite being primarily distributed in the atrioventricular and interventricular grooves (24, 25). EAT is unique among other cardiac adipose tissue depots as it shares the same embryological origin and circulation with the myocardium: both structures are derived from the splanchnic mesoderm and receive their blood supply from the coronary arteries (26). The anatomical proximity of EAT to myocardium and shared circulation has led to a growing body of evidence supporting a paracrine role of EAT in the development of cardiac disease. Although this evidence is compelling, inconsistent definitions of cardiac adiposity between studies is a limitation to the extrapolation of evidence between fat volume and arrhythmias. Pericardial fat is often used interchangeably with EAT, although the latter strictly refers to adipose tissue deep to the parietal pericardium. On the other hand, pericardial fat is the combination of EAT and adipose tissue external to the parietal pericardium, i.e., paracardial fat (Fig. 2).

EAT has a physiological role of protecting the myocardium against potential stressors. Because of its elastic and compressible properties, it protects the coronary arteries from mechanical forces generated by arterial pulsation and myocardial contraction (27). Unlike other fat depots, EAT has a uniquely higher rate of lipogenesis and lipolysis and therefore acts as a local energy store of free fatty acids (FFAs) and also as a buffer against the lipotoxic effects of excess FFAs (28, 29). It also functions as an endocrine organ secreting a range of adipokines (26, 30, 31). In this way, EAT has been shown to modulate proliferation of vascular smooth muscle cells and vascular contractility while also exhibiting antiapoptotic and antioxidant effects (32–35). Given that low levels of UCP-1 have been linked with atrial fibrillation (AF) (36), it has been postulated that EAT, by expressing UCP-1, may also protect against hypothermia-induced fatal arrhythmia (23).

## EAT AS A PATHOPHYSIOLOGICAL INFLAMMATORY SUBSTRATE

Under pathological conditions, EAT undergoes a phenotypic shift from a protective neighbor to an inflammatory substrate. For example, EAT in patients with coronary artery disease (CAD) exhibit a greater proinflammatory profile than in non-CAD counterparts (37). Both mRNA expression and protein levels of proinflammatory cytokines such as tumor necrosis factors-α (TNF-α), interleukin (IL)-6, and monocyte chemoattractant protein (MCP-1) have been shown to be upregulated, and anti-inflammatory adiponectin to be downregulated, after adjustment for concurrent hypertension, diabetes, dyslipidemia, and body mass index (BMI) (38–42). Consistent with the rise in MCP-1, histological analysis of EAT from CAD patients exhibited infiltration by M1 macrophages and CD68^+^ cells (43).

The shift toward a proinflammatory EAT secretome has also been observed in type 2 diabetes mellitus (T2DM), with a reduction in adiponectin and increase in expression of MCP-1 and CD68^+^ cells compared with nondiabetic counterparts (44). The transcriptome of EAT in T2DM patients has also been shown to primarily consist of genes coding for cytokine production and leucocyte recruitment, such as TNF-α, IL-6, and chemokine receptors CXCR1 and CXCR2 (45). By contrast, a study by Teijeira-Fernandez et al. (46) demonstrated no difference in the adipokine profiles of EAT in T2DM and non-T2DM patients. This may be explained by an older and more overweight non-T2DM cohort in which subclinical insulin resistance may have masked any differences in adipokine profiles between the two groups (46).

Pharmacotherapy in CAD and T2DM has been shown to reverse the inflammatory phenotype of EAT. In a study by Parisi et al. (47), 40 mg atorvastatin, an HMG-CoA reductase inhibitor, daily for a mean duration of 24 mo in CAD was associated with a lower EAT volume and a less inflammatory profile of the EAT secretome among patients undergoing cardiothoracic surgery. Consistent with this, in vitro application of 2 μM atorvastatin on EAT for 24 h resulted in selective suppression of proinflammatory adipokines (47). Similarly, among T2DM patients taking 25 mg pioglitazone, a thiazolidinedione, for a mean duration of 24 mo, there was reduced local expression of proinflammatory IL-1β in EAT compared with those without treatment (48). Likewise, 6-h incubation of EAT in vitro with 100 μM dapagliflozin, a reversible inhibitor of sodium-glucose cotransporter 2, resulted in decreased IL-8, CCL2, and CCL5 concentrations (49). Despite the evidence for EAT adopting a proinflammatory state in the context of cardiometabolic perturbation and its reversal with pharmacological modulation, whether this is a cause or consequence of concurrent disease processes remains contentious.

## EPICARDIAL VERSUS SUBCUTANEOUS ADIPOSE TISSUE SECRETOMES

Several studies have also demonstrated that EAT exhibits a more proinflammatory profile compared with other fat depots (50–53). Mazurek et al. (50) first demonstrated mRNA and protein levels of IL-1β, IL-6 and TNF-α were greater in EAT compared with subcutaneous adipose tissue (SAT) in paired samples harvested from CAD patients. EAT was also associated with a higher concentration of macrophages, mast cells and T lymphocytes. Similarly, Baker et al. (52) showed fivefold lower concentrations of adiponectin and threefold higher resistin concentrations, as well as a greater expression of IL-6, leptin, and CD45 in EAT compared with SAT. Although these findings are consistent with Mazurek et al. (50), EAT and SAT biopsies in this study were unpaired, i.e., harvested from different patient cohorts which may confound comparisons. Addressing this by harvesting paired EAT and SAT during cardiothoracic surgery, Fain et al. (51) showed that EAT expresses a greater concentration of inflammatory adipokines, such as IL-1β, MCP-1, leptin, and TNF-α compared with sternal, abdominal and leg SAT. Adipocytes isolated from EAT and cultured in vitro also demonstrated an adipokine imbalance that favors proinflammatory pathways (54). More recently, the inflammatory characteristic of EAT has been demonstrated to have a genetic basis with 383 genes encoding cytokine-receptor interaction, focal adhesion, and complement cascades (55). Consistent with this, EAT from overweight and obese individuals exhibited a greater expression of IL-1, IL-6, TNF-α, and IFN-γ that was not observed in SAT or serum, independent of concurrent coronary artery disease or hypertension status (56).

## OBESITY AND ARRHYTHMIC RISK

Obesity is characterized by excess adiposity and represents a chronic low-grade systemically proinflammatory state. Adipocytes hypertrophy with increasing adiposity creating a hypoxic environment and inducing genetic mutations that results in up- and downregulation of pro- and anti-inflammatory mediators, respectively (57, 58). Consistent with other examples of enhanced inflammatory state, obesity has been associated with increased risk of atrial and ventricular arrhythmias (59, 60). For example, AF risk may increase as much as 65% in individuals with BMI >30 kg/m^2^ compared with normal BMI (18.5–24.9 kg/m^2^), translating to a 4% increased risk for every unit increment in BMI (59, 60). This increased propensity for AF may be explained by the electrophysiological remodeling as a consequence of atrial volume expansion, conduction abnormalities, and increased expression of profibrotic mediators that contribute to generating a proarrhythmogenic substrate (61, 62). These changes are reversible with weight loss, as demonstrated in an obese ovine model in which 30% weight reduction reduced atrial pressure and fibrosis and improved conduction via increased expression of connexin-43 (Cx43) (10). In addition to systemic obesity, electrostructural remodeling has been shown to be modulated by localized adiposity, whereby changes were most pronounced in regions adjacent to EAT, implicating it with arrhythmogenesis (14).

## EAT AND CARDIAC ARRHYTHMIAS

Given that EAT is a metabolically active depot that is intimately related to the myocardium, there are several lines of evidence that implicate EAT with arrhythmogenesis. This was first demonstrated by Thanassoulis et al. (63) in the Framingham Heart study in which AF risk increased by almost 30% for every 1-SD increment in volume of pericardial fat. Furthermore, AF burden correlated with the pericardial fat volume; pericardial fat in persistent AF was 23% greater than in paroxysmal AF (64). A meta-analysis of 63 observational studies encompassing 352,275 participants also reported that 1-SD increment in the volume of EAT was associated with a 2.2-fold higher risk of persistent AF compared with paroxysmal AF (4). Furthermore, EAT has been demonstrated to be a better predictor of AF risk than other measures of adiposity, including BMI, body surface area, waist circumference, waist-to-hip ratio, intrathoracic fat, and abdominal VAT (4, 63–65). EAT volume has also been shown to be as much as 34% greater in patients with recurrent AF than those in sinus rhythm (66) with several other studies implicating EAT as an independent predictor of AF recurrence following radiofrequency- and cryoballoon-based ablation (66–70). Indeed, epicardial fat and pericardial fat confer comparable incremental risks of AF (31% and 30% per standard deviation increase between EAT and pericardial adipose tissue volume, respectively) (63, 71). Furthermore, EAT volume correlates with serum CRP in those with recurrent AF suggesting inflammation as the mediator between EAT and arrhythmic risk (70). These lines of evidence suggest that adipose tissue that is in direct contact with the myocardium contributes to a proarrhythmic substrate and that it may do so by paracrine mechanisms.

On the other hand, the evidence linking EAT volume with ventricular arrhythmogenesis is conflicting. For instance, EAT thickness positively correlates with the ventricular ectopy burden (72, 73) and is predictive of QT interval prolongation (defined as >450 ms), ventricular tachycardia/fibrillation risk in the context of heart failure, and ventricular tachycardia recurrence postablation (74–76). By contrast, others do not report an association between EAT volume and QTc interval (77, 78) but rather that EAT thickness better correlates with PR interval prolongation (79) and P wave, rather than QT, dispersion (80).

## DIFFERENCES IN EAT AND CARDIAC ARRHYTHMIAS BETWEEN SEXES

Epicardial adiposity is influenced by a number of factors aside from sex, such as obesity status and concurrent cardiometabolic ill health (22, 30, 81). It is therefore unsurprising that there are conflicting reports for whether EAT volume is greater in either sex. Some studies have reported higher EAT volume in females (24, 82). By contrast, it has also been shown to be as much as 29% greater in males undergoing cardiothoracic surgery compared with females (83). EAT in males from a similar cohort also revealed reduced expression of the cardioprotective adipokine adiponectin compared with females (84). Nevertheless, it is possible that differences in EAT volume between sexes may at least partly explain the higher age-adjusted lifetime AF risk in males compared with females (26.0 vs. 23.0%) (85, 86). Nevertheless, the incidence of AF in females increases postmenopause, resulting in higher burden of AF in females aged over 70 yr compared with males (74 vs. 58%) (87). Increased susceptibility to AF in this population could be mediated by an increased EAT volume after menopause and with increasing age (88, 89), specifically in the periatrial region, which may adversely alter the electrophysiological properties of the atria (90). The arrhythmogenicity associated with aging and EAT has also been demonstrated in rodents; expression of adiponectin in EAT decreases throughout the life span of female rats from 6 to 26 mo (91). It is therefore plausible that the differential hormonal profile between sexes and their changes with age, particularly in females, may contribute to arrhythmic risk.

## ELECTROPHYSIOLOGICAL CHANGES INDUCED BY EAT

In vitro studies support a paracrine effect of EAT in generating an arrhythmic substrate. Lin et al. (92) studied the electrophysiological changes in rabbit left atrial (LA) myocytes induced by either epicardial, retrosternal, abdominal adipocytes or adipocyte-conditioned media for 2–4 h. When compared with control, adipocyte-incubated myocytes demonstrated prolonged action potential duration (APD) at 90% repolarization (APD_90_), suggesting a shared arrhythmogenic potential among different fat depots. Moreover, EAT adipocyte-incubated myocytes displayed modulation of ionic currents, which included increased late sodium (*I*_Na_) and L-type calcium (*I*_CaL_) currents and decreased delayed rectifier potassium (*I*_Kr_) and inward rectifier potassium currents (*I*_K1_), all of which were consistent with prolonged APD (92). Furthermore, myocytes incubated in adipocyte-conditioned media exhibited prolonged APD, supporting a paracrine mechanism of action (92). Although this effect was modest in comparison to adipocyte-myocyte coculture, the duration of incubation with conditioned media was relatively short and may have underestimated this effect. Microscopic observation of adipocyte-myocyte coculture rarely revealed direct contact between adipocytes and myocytes suggesting that a paracrine mechanism of action underpinning electrophysiological modulation was more likely than an electrotonic interaction between the two cell types. A similar APD-prolonging effect in rabbit LA myocytes was observed in the context of heart failure (HF) whereby incubation of LA myocytes with HF EAT resulted in early and delayed afterdepolarizations, as well as increased spontaneous activity (93). Ventricular myocytes incubated with EAT also demonstrated APD prolongation at 90% (APD_90_), 50% (APD_50_), and 20% (APD_20_) repolarization compared with cells not exposed to EAT, in addition to having a more positive resting membrane potential whereby threshold for depolarization is reached earlier and facilitating triggered activity (94). Furthermore, the inhomogeneous distribution of EAT around the myocardium may result in APD heterogeneity and thereby generate an arrhythmogenic substrate enabling reentry (94).

More recently, Nalliah et al. (95) showed a differential effect between murine pericardial versus subcutaneous fat on cell electrophysiology in vitro. HL-1 atrial cardiomyocytes incubated with pericardial fat-conditioned media for 24 h recorded slower conduction velocities and longer activation times than cells exposed to inguinal SAT. Analyzes of their respective secretomes showed that pericardial fat had an abundance of proteins associated with disruption of intermyocyte adhesion, modulation of cellular metabolism, and inflammation compared with the relatively anti-inflammatory proteome of inguinal SAT (95). This was consistent with observations of connexin40 lateralization in cells incubated with pericardial versus subcutaneous fat and explains the conduction slowing observed in the study. The relatively proinflammatory proteome of pericardial fat is also consistent with the paracrine effect of cardiac adiposity in arrhythmogenesis.

Electrotonic interactions between adipocytes and myocytes may also alter electrophysiological function of the myocardium. For example, Ten Sande et al. (96) showed that monolayers of neonatal rat ventricular myocytes incubated with human and rat adipose-derived stromal cells exhibited slower conduction than cells treated with their respective conditioned media. However, given that adipose tissue includes adipocytes and stromal cells, both of which synthesize and secrete adipokines, it is possible that conditioned media from the stromal cell fraction alone may underestimate the paracrine effect of whole adipose tissue. Interestingly, porcine adipose-stromal cell conditioned media exerted a similar degree of conduction slowing as the stromal-adipocyte coculture in the same experimental settings, supporting a species-specific adverse paracrine effect of adipose tissue on adjacent myocardium (96).

## MECHANISMS UNDERLYING ADVERSE ELECTROPHYSIOLOGICAL REMODELING DUE TO EAT

### Ion Channel Modulation by Cytokines

**Figure 3.**
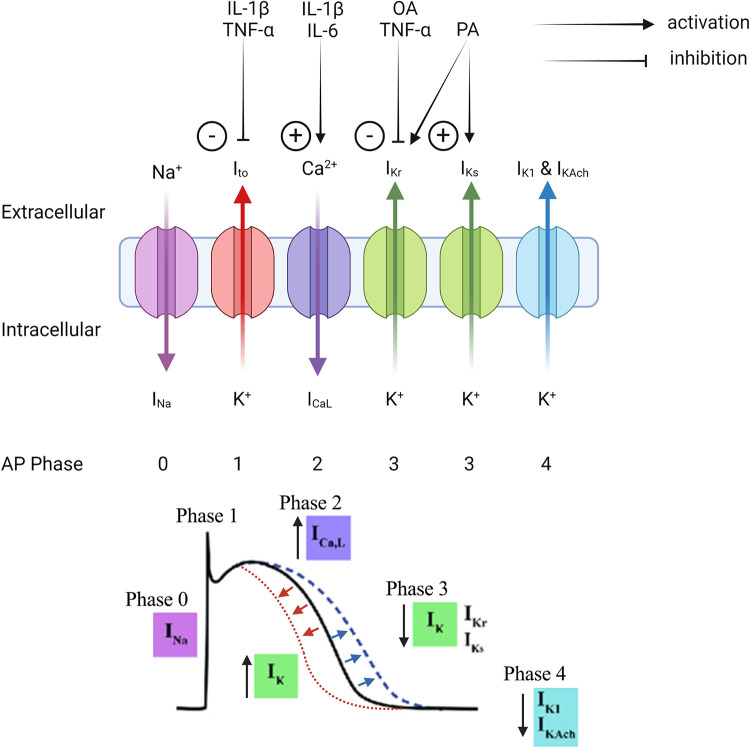
Hypothetical mechanisms by which epicardial adipose tissue (EAT)-derived inflammatory cytokines and free fatty acids modulate ion channels resulting in action potential (AP) duration shortening and prolongation. EAT secretes IL-1β, TNF-α, IL-6, oleic acid (OA), and palmitic acid (PA), which exert a paracrine effect on the ion channels expressed in the membranes of myocardial cells. The activation of L-type calcium channels (*I*_CaL_) and inhibition of fast (*I*_Kr_) and slow (*I*_Ks_) rectifying potassium channels results in action potential prolongation (dashed blue line). By contrast, activation of the rectifying potassium currents by PA shortens action potential duration (dashed red line). *I*_Na_, sodium current; *I*_K1_, inward rectifier potassium current; *I*_to_, transient outward potassium current; *I*_KAch_, acetylcholine-activated inward-rectifying potassium current. Created with BioRender.com, and published with permission.

**Figure 4.**
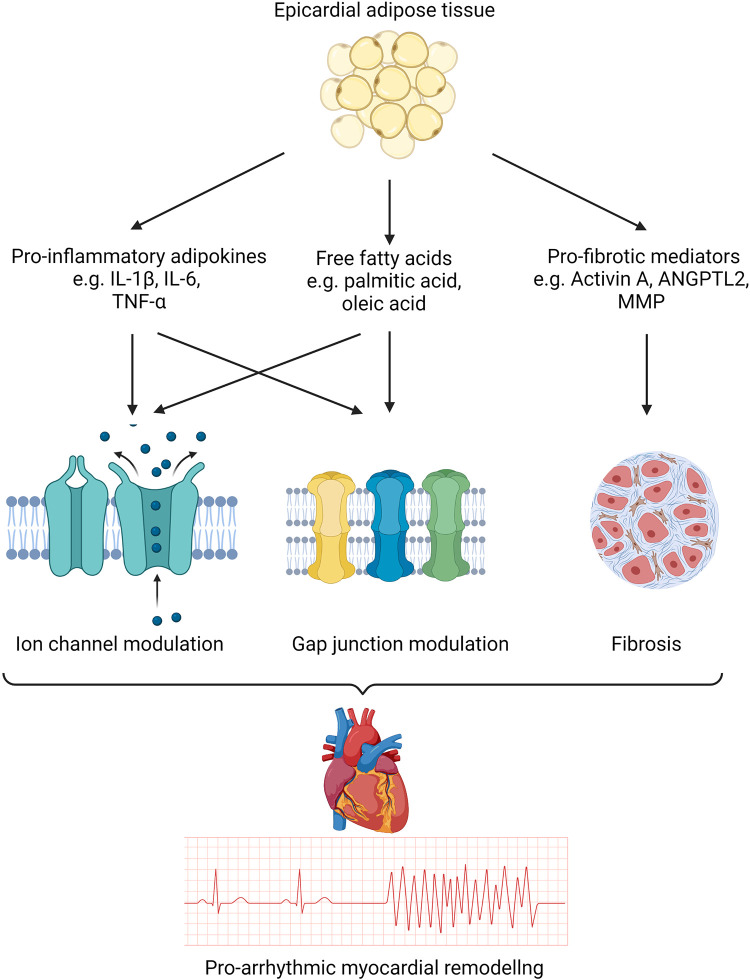
Mechanisms underpinning electrophysiological remodeling associated with epicardial adipose tissue (EAT). EAT is a rich source of proinflammatory cytokines and free fatty acids that modulate ion channels and gap junction proteins. Additionally, profibrotic mediators have been shown to induce structural remodeling. These mechanisms result in delayed conduction and repolarization heterogeneity within the myocardium that renders it vulnerable to arrhythmias. ANGPTL2, angiopoietin-like 2; MMP, matrix metalloproteinase. Created with BioRender.com, and published with permission.

Given the considerable evidence supporting local and systemic inflammation in inducing a proarrhythmic substrate (97–106), several in vitro studies have demonstrated how cytokine-mediated ion channel modulation may underpin this phenomenon (Fig. 3). Although much of this evidence is not derived from the context of EAT, cytokines that are known to be actively secreted by EAT, such as IL-1β, TNF-α, and IL-6, have been shown to alter ion channel function and may mediate part of the increased arrhythmogenicity associated with increasing adiposity (56) (Fig. 4).

For instance, IL-1β causes APD_90_ prolongation that may be explained by increased *I*_Ca2+_ current (107). Reduced transient outward potassium current (*I*_to_) and altered Ca^2+^ homeostasis in the sarcoplasmic reticulum that increases Ca^2+^ spark and prolongs APD have been shown to increase propensity for spontaneous depolarization and triggered activity (108). TNF-α and IL-1β synergistically increase sarcoplasmic reticulum (SR) Ca^2+^ leak and reduce SR Ca^2+^ content, which increases the frequency of spontaneous Ca^2+^ waves (109). Transgenic mice overexpressing TNF-α exhibit elevated diastolic Ca^2+^ concentration and a prolonged Ca^2+^ transient, resulting in slow heterogenous conduction and consequently a functional line of conduction block (110). IL-6 treatment in guinea pig ventricular myocytes has also been shown to alter APD, an effect that was reversed with an antagonist (111).

However, much of the evidence for cytokine-mediated ion channel modulation comes from murine models. Although the currents that contribute to rapid depolarization (*phase 0*) and resting membrane potential (*phase 4*), *I*_Na_ and *I*_K1_, respectively, are common among mice and humans, those determining AP morphology differ significantly. *I*_CaL_ is weaker in murine cardiomyocytes resulting in an almost absent plateau phase (*phase 2*) (112, 113). In mice, repolarization is driven by *I*_to_, *I*_K,slow_ and a noninactivating steady-state current (*I*_ss_), whereas in humans it is primarily mediated by the rapid and slow delayed outward rectifier potassium currents (*I*_Kr_ and *I*_Ks_) (112–114). Potassium currents equivalent to *I*_K,slow_ and *I*_ss_ have not yet been identified in the human ventricles, and *I*_Kr_ and *I*_Ks_ have minimal roles in the murine cardiomyocytes. Thus, murine studies showing APD prolongation via modulation of *I*_K,slow_ would not be expected to be replicated in human cardiomyocytes. The effect of cytokines on *I*_Kr_ and *I*_Ks_ is also less well characterized (113, 115). This may limit the extrapolation of the effects of EAT on ion channel function in humans.

### Ion Channel Modulation by Free Fatty Acids

In addition to inflammatory cytokines, FFAs secreted by EAT exert an arrhythmogenic influence on the underlying myocardium via direct modulation of APD (Fig. 4). EAT, like other fat depots, is a rich source of saturated, monounsaturated, and polyunsaturated FFAs, with a greater content of eicosapentanoic and arachidonic acids compared with SAT (116). In contrast to the APD-prolonging effect of cytokines, FFAs have been shown to have inconsistent effects on APD duration. Aromolaran et al. (117) demonstrated that high-fat-diet (HFD) in guinea pigs shortened APD_90_ and APD_30_ and suggested that some of this APD shortening may be mediated by FFAs secreted from EAT. Whole cell patch clamp of atrial myocytes showed that 1-h incubation with palmitic acid (PA), a saturated FFAs, enhanced both *I*_Kr_ and *I*_Ks_, which reduced APD_90_ and APD_30_ by 46 and 30%, respectively (117). Haim et al. (118) showed similar results in murine ventricular myocytes exposed to PA recorded *I*_to_ and *I*_Ks_ currents up to 20% greater than control, resulting in APD_90_, APD_50_, and APD_20_ shortening. These effects were reversible with pharmacological antagonism of the potassium channels consistent with FFA-induced augmentation of repolarizing currents. O’Connell et al. (119) similarly demonstrated shortened APD_30_ in ovine LA myocytes after 24-h exposure to PA, although this did not alter APD_50_ or APD_80_. Optical imaging of LA myocytes incubated with stearic acid (saturated FFA) revealed altered T-tubular architecture and reduced *I*_CaL_, perhaps explaining APD shortening.

When compared with the shortening of APD observed with PA, studies have shown inconsistent effects of unsaturated FFA oleic acid (OA) on APD. For instance, incubation of atrial myocytes with OA in one study showed reduced *I*_Kr_ functionality and consequently prolonged APD_90_ and APD_30_ by 14 and 42%, respectively (117). On the other hand, Lin et al. (120) showed 24-h exposure to OA shortened APD_90_ and APD_50_ in HL-1 myocytes and reported higher incidence of delayed afterdepolarization compared with control cells. These electrophysiological changes were attributed to intracellular dysregulation of calcium and sodium ions, reflected by larger calcium transients and calcium stores in the sarcoplasmic reticulum, as well as attenuated sodium-calcium exchanger and sodium-potassium pump currents. Consistent with the ionic current modulation, OA-treated cells displayed higher expression of sarcoplasmic reticular Ca^2+^-ATPase and calmodulin kinase II but lower expression of the sodium-potassium ATPase than control myocytes (120).

More recently, Aromolaran (121) showed that PA- and OA-induced changes in *I*_to_ and *I*_Kr_ in guinea pigs can be attenuated with the inhibition of phosphoinositide 3-kinase (PI3K) and serine-threonine protein phosphatase-2 (PP2A), respectively. In addition to this, dietary fish oils have shown to prevent electrical remodeling and hence susceptibility to triggered activities via modulation of the potassium currents (122, 123). This had the additional benefits of preventing structural remodeling in volume overload and heart failure, which could have an antiarrhythmic effect. Therefore, the current body of evidence suggests possibilities of therapeutically treating and preventing electrophysiological changes that are induced by lipotoxicity and obesity.

Although these studies provide insights into the modulation of ionic currents in a lipotoxic environment, the contrasting effects of FFA on APD may stem from differences in animal models, including atrial versus ventricular myocytes, as well as the duration of FFA exposure. Furthermore, despite the opposing effects of cytokines and FFAs, both APD prolongation and shortening facilitate reentry and delayed afterdepolarizations, respectively. It is therefore likely that EAT-derived cytokines and FFAs contribute to spatial heterogeneities of repolarization, although the predominant mechanism underpinning arrhythmogenesis in epicardial obesity remains to be elucidated.

### Gap Junction Modulation

In addition to ionic modulation, inflammatory mediators and FFAs have been associated with the alteration of gap junctions (Fig. 4). Gap junctions are primarily located at the intercalated disks and facilitate electrical coupling between adjacent cardiomyocytes. They consist of two hemichannels or connexons, each of which is formed by six ion-channel proteins called connexins. Connexin40 (Cx40), -43 (Cx43), and -45 (Cx45) are the most common in the human heart. These are primarily found in gap junctions in the atrial, ventricular, and specialized conducting tissue.

The relationship between altered connexin expression and propensity for atrial and ventricular arrhythmias is well established (124–126). More recent studies have demonstrated a relationship between connexin expression and BMI. For example, weight loss of 30% in obese ovine model has been associated with increased expression of Cx43 and concurrent improvements in conduction velocity, decreased conduction heterogeneity, and reduced AF susceptibility (10). Given that obesity and BMI correlate with EAT volume (127), it is possible that this phenomenon is the consequence of EAT attenuation. Indeed, Egan Benova et al. (128) showed that weight gain and an increase in EAT volume following high-sucrose diet was associated with downregulation of Cx43 and greater inducibility of ventricular arrhythmias. Chronic HFD also alters connexin distribution from intercalated disks to the longitudinal membrane of cardiomyocytes, and this “lateralization” was shown to correlate with higher EAT volume and slower conduction velocity (95, 129).

Cytokines secreted by EAT, such as IL-6 and TNF-α, have been shown to cause gap junctional changes in experimental settings. For instance, Lazzerini et al. (130) demonstrated an inverse relationship between circulating concentrations of IL-6 protein, and Cx40 and Cx43 mRNA in patients undergoing cardiothoracic surgery. Moreover, increasing concentrations of IL-6 were associated with greater P wave dispersion, a predictor of AF (131). An in vitro study showed reduced Cx40 and Cx43 expression in HL-1 atrial myocytes incubated with IL-6 for 24–48 h, an effect that was reversed by preincubation with monoclonal antibody against IL-6 (130). Transgenic mice overexpressing TNF-α also demonstrated Cx40 downregulation that is associated with slower atrial conduction and increased incidence of supraventricular arrhythmias (132). Although a 90-min exposure of TNF-α in guinea pig heart did not suppress Cx43 protein expression, it was nonetheless associated with its distribution away from intercalated disks (132, 133). Furthermore, TNF-α exposure altered ephaptic coupling, in which conduction relies on the close spatial arrangement of adjacent cardiomyocytes and high density of sodium channels in the sarcolemma (133). Conduction via this mechanism occurs as depolarization of myocyte sequesters sodium ions in the cleft, rendering the voltage of the cleft more negative and creating a steep voltage gradient between the adjacent myocytes. This stimulates the opening of sodium channels in the adjacent myocyte allowing propagation of electrical activity from one myocyte to the other. In this way, narrow perinexi enable cardiomyocytes to remain electrically coupled in instances where gap junction coupling may be compromised (134, 135). TNF-α has been shown to widen the perinexus between adjacent cardiomyocytes and consequently reduce ephaptic coupling resulting in slower conduction velocity (133). Indeed, perinexal width has been shown to be greater in patients with AF (136). Along these lines, other inflammatory proteins such as vascular endothelial growth factor also disrupt gap junctions resulting in conduction slowing predisposing normal hearts to arrhythmias (137). Langendorff-perfused guinea pig heart preparations have also been used to investigate the effects of perinexal width variation on arrhythmia susceptibility and how this may be modulated by extracellular sodium and calcium concentrations experimentally (138–140). It is therefore possible that the inflammatory secretome of EAT may exert proarrhythmic effects by modulating both gap junction and ephaptic coupling.

FFAs also modulate connexin expression with OA and arachidonic acid shown to inhibit gap junctional coupling within hours of exposure in neonatal rat cardiac myocytes (141, 142). This was rapidly reversed with washing the cells in a FFA-free solution, suggesting the effect is most likely to be due to an extracellular component activating membrane receptors (141, 142) and subsequently initiating an intracellular signaling pathway mediated by protein kinases via G protein-coupled receptors (143, 144). A 24-h incubation with OA has been shown to activate protein kinase C in rat cardiomyocytes which consequently induced posttranslational phosphorylation of connexins and hence the disintegration of gap junctions (145). Furthermore, protein kinase A, PI3K, AKT, Src, and MEK1/2 have been shown to similarly modulate connexin functionality following FFA exposure (146–149). FFA-activated protein kinases also modulate adherens junctions (150), which comprise catenin and cadherin proteins that maintain the integrity of intercalated disks (151). Neonatal rat cardiomyocytes treated with OA result in activation of the PKCα/PKCɛ-Fyn signaling cascades and consequently tyrosine phosphorylation of β-catenin and p120 catenin (150). This in turn results in decreased binding of β-catenin with α-catenin and p120 catenin with *N*-cadherin, causing adherens junctions to become unstable and gap junction disassembly because of disruption of catenin-ZO-1-Cx43 complex (152).

### Fibrotic Remodeling

Fibrotic remodeling in the myocardium provides a substrate for reentry as it produces tortuous paths of conduction and concurrently slows macroscopic conduction through the myocardium. Mahajan et al. (62) demonstrated atrial fibrosis increases with adiposity and provided evidence for EAT as a source of profibrotic cytokines that are responsible for structural remodeling. Indeed, Nalliah et al. (95) also demonstrated the association between EAT volume and degree of myocardial fibrosis and Abe et al. (153) provided histological evidence of a greater degree of fibrosis in LA adjacent to EAT compared with tissue that was not associated with EAT (16.5 vs. 8.4%) and that this may be explained by paracrine mechanisms . Severity of atrial fibrosis positively correlated with EAT fibrosis, meanwhile fibrotic EAT was infiltrated with inflammatory macrophages that were positioned between the adipocytes and the myocardium, suggesting involvement of local inflammation in the induction of fibrotic remodeling (153). IL-1β, IL-6, TNF-α, and MCP-1, which are actively secreted by EAT, have been associated with atrial collagen deposition and the degree of atrial fibrosis was positively correlated with fibrotic markers such as platelet-derived growth factor-BB and matrix metalloproteinase (MMP)-2 (153). Angiopoietin-like 2 (ANGPTL2), which is also found in EAT, also positively correlated with the local concentrations of MMP2 and MMP9, as well as atrial collagen level, implicating its mediation of myocardial fibrosis (153, 154). Other studies have demonstrated that MMP2 and MMP7 can induce interstitial fibrosis and that MMP2 is positively correlated with EAT volume as well as AF severity (155, 156). Moreover, connective tissue growth factor (cTGF) is more highly expressed in EAT of AF patients compared with those in sinus rhythm (157). Local concentrations of cTGF also strongly correlated with atrial fibrosis and were an independent predictor of AF occurrence, suggesting EAT may be the source of cTGF facilitating atrial remodeling (157).

Activin A, a member of the transforming growth factor-β (TGF-β) superfamily, is also abundantly synthesized in EAT and has been associated with fibrotic remodeling of the atria (158). Organo-culture of rat atria treated with activin A for a week at a concentration of 2 ng/mL resulted in increased production of collagen types I, III, and VI and marked global fibrosis (158). Furthermore, preincubation with anti-activin A antibodies prevented fibrotic remodeling induced by EAT-conditioned media. EAT-conditioned media and activin A induced comparable changes in atrial gene expression, specifically increased TGF-β1 and TGF-β2, suggesting that EAT-induced atrial fibrosis in this study was primarily facilitated by activin A (158).

### Fatty Infiltration

Aside from paracrine effects, epicardial adipocyte infiltration into the myocardium may separate myocardial fibers resulting in conduction slowing or block that would facilitate reentrant arrhythmias. This is not dissimilar to the fibro-fatty infiltration observed with inherited cardiomyopathies (159). For example, Venteclef et al. (158) showed that myocardial fibrosis was more profound in areas of EAT filtration into atrial and ventricular tissue in vitro, and that this occurred at the interface between adipocytes and the surrounding myocardium as well as neighboring tissue. These effects were unique to EAT and were not observed with SAT. Mahajan et al. (62) showed EAT infiltration into the posterior left atria of chronically obese sheep was associated reduced endocardial voltage. In addition to conduction slowing, greater conduction heterogeneity and fractionated electrograms, the atria of obese sheep were also more dilated and pressure overloaded compared with their nonobese counterparts (62). Similar conduction abnormalities associated with obesity have been shown to colocalize with EAT in humans and may be attributed to atrial fatty infiltration (14). As well as greater EAT volume, atria of obese adults exhibit a global reduction in conduction velocity, greater electrogram fractionation, and reduced voltage, which correlate more strongly with EAT volume than global measures of obesity, such as BMI. (14) Vulnerability for AF as a consequence of epicardial fatty filtration has also been demonstrated in obese canines with or without rapid atrial pacing to induce AF (160). Interestingly, epicardial fatty infiltration occurred following rapid atrial pacing to induce AF, even in nonobese animals, and correlated with interstitial fibrosis (160). A lower negative resting membrane potential, smaller action potential amplitude, and longer repolarization time as well as augmented transient outward potassium current and reduced L-type calcium and inward rectifier potassium currents have all been observed with cardiomyocytes connected to epicardial adipose tissue and may explain the vulnerability for arrhythmias as a consequence of fatty infiltration (94).

## WEIGHT REDUCTION REDUCES EAT VOLUMES AND REVERSES PROARRHYTHMIC REMODELING

Given the evidence supporting EAT as a source of a proarrhythmic secretome, it follows that its regression may reduce arrhythmic risk. EAT volume has been shown to decrease with weight reduction achieved by lifestyle modification and surgical intervention. The EISNER (Early Identification of Subclinical Atherosclerosis by Noninvasive Imaging Research) registry demonstrated an association between weight loss and EAT volume, such that a 2.3% reduction in EAT was recorded among participants achieving >5% body weight loss (161). However, <5% whole body weight loss was not associated with significant changes in EAT volume (162). Specific weight loss programs via low-calorie diet targeted at obese patients also demonstrated similar pattern of EAT change (81, 163). Iacobellis et al. (81) showed that a 6-mo 900 kcal/day diet resulted in 20% whole body weight loss and reduced EAT thickness by 32%. Kim et al. (163) showed a more modest calorie restriction of 1547 kcal/day over 12 wk was sufficient to result in significant changes in body weight (−11.0%) and EAT thickness (−17.2%) as well as visceral abdominal adiposity. An hour of aerobic exercise achieving 60–70% of maximal predicted heart rate three times a week for 12 wk in obese individuals has been shown to result in almost 9% reduction in EAT thickness (164). Weight loss following bariatric surgery has been shown to regress EAT volumes by as much as 24%, although results may vary according to intervention (14.6% with Roux-en-Y gastric bypass vs. 5.3% with sleeve gastrectomy) (165, 166).

Correspondingly, recent studies have demonstrated reduced propensity for arrhythmias following weight loss. In a randomized controlled trial Abed et al. (167) showed calorie restriction and low intensity exercise resulted in a greater weight reduction than lifestyle advice over a median follow-up of 15 mo (14.3 kg vs. 3.6 kg). More importantly, patients restricting calorie intake and undertaking exercise recorded lesser burden and severity of AF than those receiving lifestyle advice (167). The reduction in arrhythmia burden has been reported up to 2 yr following AF ablation; cardiometabolic risk factor management was associated with arrhythmia-free survival in 32.9 and 87% of patients undergoing single and multiple ablations, respectively, compared with 9.7 and 17.8% in those receiving standard care (168). Long-term monitoring of weight managed AF patients in the LEGACY (Long-Term Effect of Goal-Directed Weight Management in an Atrial Fibrillation Cohort) study showed a dose-response relationship between weight loss and arrhythmia-free survival; >10% weight loss was associated with a sixfold greater likelihood of arrhythmia-free survival than those recording <10% weight loss (169). These effects were more marked in those achieving consistent and progressive weight reduction and were partly reversed with weight gain. A fluctuation in body weight of >5% following lifestyle modification conferred a twofold higher risk of AF (169). The same cohort of patients in the LEGACY study were subsequently investigated in the REVERSE-AF (PREVEntion and regReSsive Effect of weight-loss and risk factor modification on Atrial Fibrillation) study to assess the progression and possible reversal of AF with sustained weight loss (11). The study reported 88% of patients with >10% weight loss reversed from persistent to paroxysmal AF, compared with 49 and 26% in groups achieving 3–9% and <3% weight loss, respectively (11). Therefore, given that EAT volume regresses with weight reduction, lifestyle modification represents a noninvasive and inexpensive intervention by which arrhythmias and its risk can be managed.

Despite such compelling evidence for weight reduction for the management of AF, it is unclear whether similar benefits are conferred for ventricular arrhythmias. A meta-analysis comprising 7,197 patients demonstrated prolonged QT interval and QT dispersion in overweight and obese individuals compared with normal weight and that this reversed by 25.77 ms and 13.47 ms with weight loss (170). Given that QT interval and QT dispersion are metrics by which ventricular arrhythmic risk is determined (171–175), it is plausible that the benefits of weight loss may extend to arrhythmias of ventricular origin, although this remains to be determined.

## CONCLUSIONS

Given that the association between adiposity and arrhythmic risk is established, there has been increasing interest in understanding the mechanisms underlying this relationship and the interventions by which the risk can be mitigated. EAT is a rich source of proinflammatory cytokines and FFA, which, owing to its anatomical proximity, renders the myocardium vulnerable to arrhythmias via paracrine mechanisms that modulate ionic currents and gap junctions and induces fibrotic remodeling. These effects are, however, reversible with aggressive risk factor management and lifestyle modification. Although much of this evidence comes from studying AF, in vitro studies suggest that similar mechanisms also underpin a higher risk ventricular arrhythmia in obesity. Prospective clinical studies exploring these effects and those of reverse remodeling following weight reduction are required to affirm these findings.

## GRANTS

This work was supported by British Heart Foundation Grant RG/16/3/32175 (to F. S. Ng).

## DISCLOSURES

No conflicts of interest, financial or otherwise, are declared by the authors.

## AUTHOR CONTRIBUTIONS

K.H.K.P., T.H., and C.S.L. conceived and designed research; K.H.K.P., T.H., and C.S.L. prepared figures; K.H.K.P., T.H., and C.S.L. drafted manuscript; K.H.K.P., T.H., and F.S.N. edited and revised manuscript; K.H.K.P., T.H., C.S.L., and F.S.N. approved final version of manuscript.
